# The Impact of Muscle Fatigue on McArdle Disease: A Case Report

**DOI:** 10.7759/cureus.103340

**Published:** 2026-02-10

**Authors:** Leandro Valente, Gabrielle Santos, Ricardo Velho, José Filipe Santos, Odete Duarte, Lurdes Correia

**Affiliations:** 1 Internal Medicine Department, Centro Hospitalar e Universitário de Coimbra, Coimbra, PRT

**Keywords:** creatine kinase elevation, glycogen storage disease type v, mcardle’s disease, metabolic diseases, myophosphorylase deficiency

## Abstract

McArdle disease (McA) is a rare metabolic disorder of autosomal recessive inheritance caused by pathogenic variants in the PYGM gene, which lead to a deficiency of the myophosphorylase enzyme. This enzymatic defect impairs muscle glycogenolysis, typically resulting in exercise intolerance, premature fatigue, and exertional cramps triggered by anaerobic or high-intensity physical activity starting in childhood or adolescence. However, the diagnosis is frequently delayed due to the heterogeneous and non-specific presentation of these symptoms.

The authors report a case of a 61-year-old woman with a lifelong history of exercise intolerance and disproportionate muscle fatigue that restricted her physical activity since her youth. She presented with persistent, idiopathic elevations of creatine kinase (CK) over several years. The patient had no history of myoglobinuria and showed preserved renal function and no evidence of acute rhabdomyolysis, despite marked hyperCKemia. Cardiac involvement was also excluded. After excluding more common secondary causes of hyperCKemia, such as statin-induced myopathy and inflammatory conditions, the persistence of marked hyperCKemia and specific exercise-induced symptoms suggested a metabolic myopathy, such as McArdle disease. Molecular analysis was performed, identifying the homozygous pathogenic variant c.280C>T (p.Arg94Trp) in the PYGM gene and confirming the diagnosis of McArdle disease.

## Introduction

McArdle disease, also known as glycogen storage disease type V, is caused by partial or complete deficiency of the glycogen phosphorylase enzyme (GP), also called myophosphorylase, due to mutations in the PYGM gene (which encodes the myophosphorylase enzyme) on chromosome 11q13 [1,2]. This deficiency blocks the initial step of glycogenolysis, the process that breaks down stored glycogen into glucose-1-phosphate in muscle cells [3]. Consequently, muscles are unable to use their vast glycogen reserves as an immediate energy source during anaerobic exercise. This leads to an acute energy crisis within the muscle fibers, resulting in exercise intolerance [4].

First described in 1951 by Dr. Brian McArdle, this condition presents with symptoms of easy fatigue and muscle cramps, mainly occurring during the first 10 minutes of high-intensity or anaerobic exercise (such as lifting heavy weights or sprinting) and typically improving with rest [5]. Approximately 50% of patients experience recurrent episodes of myoglobinuria, although reported rates vary across different clinical cohorts. Symptoms usually appear in the first decades of life, although the diagnosis often remains unknown until later in life [6].

Laboratory findings often reveal elevated resting creatine kinase (CK) levels [4]. While glycogen phosphorylase isoforms exist in various tissues, the myophosphorylase isoform (encoded by the PYGM gene) is specific to skeletal muscle. Consequently, the clinical phenotype of McArdle disease is exclusively limited to skeletal muscle involvement, as the brain and liver isoforms remain functional. Mutations in the GP gene at 11q13 inactivate the enzyme, and at least 179 pathogenic variants affecting PYGM have been reported to date, with new mutations continuing to be identified as genetic screening expands [7]. Consequently, myophosphorylase deficiency results in the accumulation of glycogen in tissues [3].

The reported prevalence of McA varies but is estimated at approximately one in 100,000 individuals [8]. In Portugal, McA prevalence is unknown, as only a small number of cases have been reported [9]. Most cases manifest during the second or third decades of life. In current clinical practice, the diagnostic approach has shifted significantly. While muscle biopsy was traditionally used to demonstrate enzyme deficiency, non-invasive genetic testing is now the preferred first-line diagnostic tool. Muscle biopsy is increasingly reserved for selected cases where genetic results are inconclusive [10]. The authors report a case of a 61-year-old woman who was diagnosed with McArdle disease after ruling out other CK-elevation-related conditions and genetic testing.

## Case presentation

A 61-year-old woman with a personal history of hypertension, dyslipidemia, and degenerative osteoarticular disease, on chronic medication with pitavastatin 2 mg once daily, amitriptyline 10 mg once daily, carvedilol 25 mg half tablet twice daily, lercanidipine 20 mg once daily, and candesartan 32 mg once daily, presented to the emergency department (ED) with generalized malaise and a feeling of overall weakness upon waking. The symptomatology had been long-standing, but the patient noticed an exacerbation on the day of ED admission. She had a normal physical and neurological examination. Laboratory results revealed elevated CK of 5609 U/L (normal value: <171 U/L) during the initial assessment, lactate dehydrogenase (LDH) of 420 U/L (normal value: <248 U/L), aspartate aminotransferase (AST) of 83 U/L (normal value: <35 U/L), and alanine aminotransferase (ALT) of 53 U/L (normal value: <45 U/L), with normal renal function, effectively ruling out acute kidney injury related to rhabdomyolysis. High-sensitivity troponin I was also normal, which, combined with a normal electrocardiogram, effectively excluded acute cardiac etiology. The laboratory parameters are summarized in Table 1. The urinalysis showed no abnormalities, and the electrocardiogram was normal. Given the patient’s clinical stability, the absence of myoglobinuria on urinalysis, and the maintenance of normal renal function, she was managed with intravenous hydration and deemed suitable for outpatient follow-up on internal medicine consultation. The lack of systemic complications or acute kidney injury provided the rationale for avoiding hospitalization despite the marked CK elevation. Notably, the patient reached a peak CK level of 5609 U/L on the day of admission, with subsequent stabilization during follow-up.

During the outpatient diagnostic workup, a transthoracic echocardiogram was performed, which revealed normal biventricular function and no significant valvular disease. Additionally, there was no clinical or imaging evidence of chronic pulmonary disease, effectively ruling out primary cardiopulmonary etiologies for her exercise intolerance before proceeding to more specific metabolic investigations.

Subsequent tests showed persistently elevated CK levels ranging between 252 and 3062 U/L, with normalization of the LDH, AST, and ALT. The patient had no previous CK values available for comparison in her medical records. Serum myoglobin levels were normal, and urinalysis remained normal, including the absence of myoglobinuria. Autoimmunity testing was unremarkable, including antinuclear antibodies, anti-SSA, anti-SSB, and anti-Sm.

**Table 1:** Patient's serum chemistry.

Laboratory parameters	Values	Reference range
Creatinine (mg/dL)	0.82	0.55-1.02
Creatine kinase (U/L)	5609	<171
Lactate dehydrogenase (U/L)	420	<248
Aspartate aminotransferase (U/L)	83	<35
Alanine aminotransferase (U/L)	53	<45
Alkaline phosphatase (U/L)	90	30-120
Gamma-glutamyl transferase (U/L)	21	<38
Total bilirubin (mg/dL)	0.6	0.2-1.2
High-sensitivity troponin I (ng/L)	<1.9	<1.9
Myoglobin (ng/mL)	62	9-82
Aldolase (U/L)	8.6	<7.6
C-reactive protein (mg/dL)	0.27	<0.50

The patient described a history of unexplained easy muscle fatigue since childhood, which limited her participation in school activities. While she did not feel significantly limited in her daily life, she noticed that her fatigue was disproportionate to the physical exertion involved and that recovery occurred after a short period of rest (less than 10 minutes), allowing her to resume activities, a pattern compatible with the "second wind" phenomenon often associated with this condition. She denied other symptoms, such as weight loss, night sweats, and chest pain. Family history was clinically significant, as several close relatives exhibited similar symptoms of exercise intolerance. Notably, one of her brothers was unable to complete his military service due to premature muscle fatigue. Although these relatives had never undergone formal etiological studies, their clinical presentations were highly suggestive of a shared hereditary metabolic myopathy.

During outpatient follow-up, other causes of elevated CK levels were excluded. Statin-induced myopathy was considered unlikely as her symptoms dated back to childhood, decades before the initiation of pitavastatin, and persisted despite the low dosage of the medication.

Given the long history of symptoms and the presence of similar symptoms in direct relatives, genetic testing for muscular diseases was performed after obtaining informed consent. The homozygous c.280C>T (p.Arg94Trp) variant in the PYGM gene was identified, establishing the definitive diagnosis of McArdle disease.

## Discussion

The pathophysiology of McArdle disease is clinically restricted to skeletal muscles, where the lack of a functional myophosphorylase enzyme prevents the breakdown of stored glycogen during anaerobic or high-intensity isometric exercise, where the sudden demand for glucose-1-phosphate cannot be met due to the enzymatic block [11]. The absence of a functional myophosphorylase enzyme means that the patient's muscles cannot break down stored glycogen to produce energy, forcing them to rely on alternative, less efficient metabolic pathways [8].

This acute energy deficit within muscle cells is the direct cause of the exercise intolerance, muscle cramps, and myalgia [12]. While the patient in this case did not experience myoglobinuria, it's a significant clinical feature observed in up to half of McArdle patients, highlighting the potential for severe rhabdomyolysis and acute kidney injury following vigorous physical activity [13].

Diagnosis is most commonly made in the second or third decade of life [14]. However, as in the case described here, it is very common for patients to perceive their symptoms as part of everyday life, delaying detailed etiological investigations. The long-standing nature of the patient's symptoms, which began in childhood, combined with the lack of previous CK values for comparison, contributed to the diagnostic challenge. Also, the lack of a diagnosis in the patient's close relatives, who presented with similar symptoms, highlights a significant factor contributing to the diagnostic delay of this genetic disease. This clinical case exemplifies the most common manifestation of the disease, with symptoms of generalized muscle weakness, exercise intolerance, and cramps beginning in childhood. A particularly relevant feature, and a key diagnostic sign, was the patient's description of rapid recovery after brief periods of rest, a pattern consistent with the "second wind" phenomenon [2].

While some diagnostic tests, such as an electromyography, can suggest McArdle disease, the definitive diagnosis is established through genetic testing, which is currently the preferred first-line approach. Muscle biopsy for the determination of myophosphorylase activity remains a valuable diagnostic tool but is increasingly reserved for cases where genetic results are inconclusive or unavailable [7].

**Figure 1:**
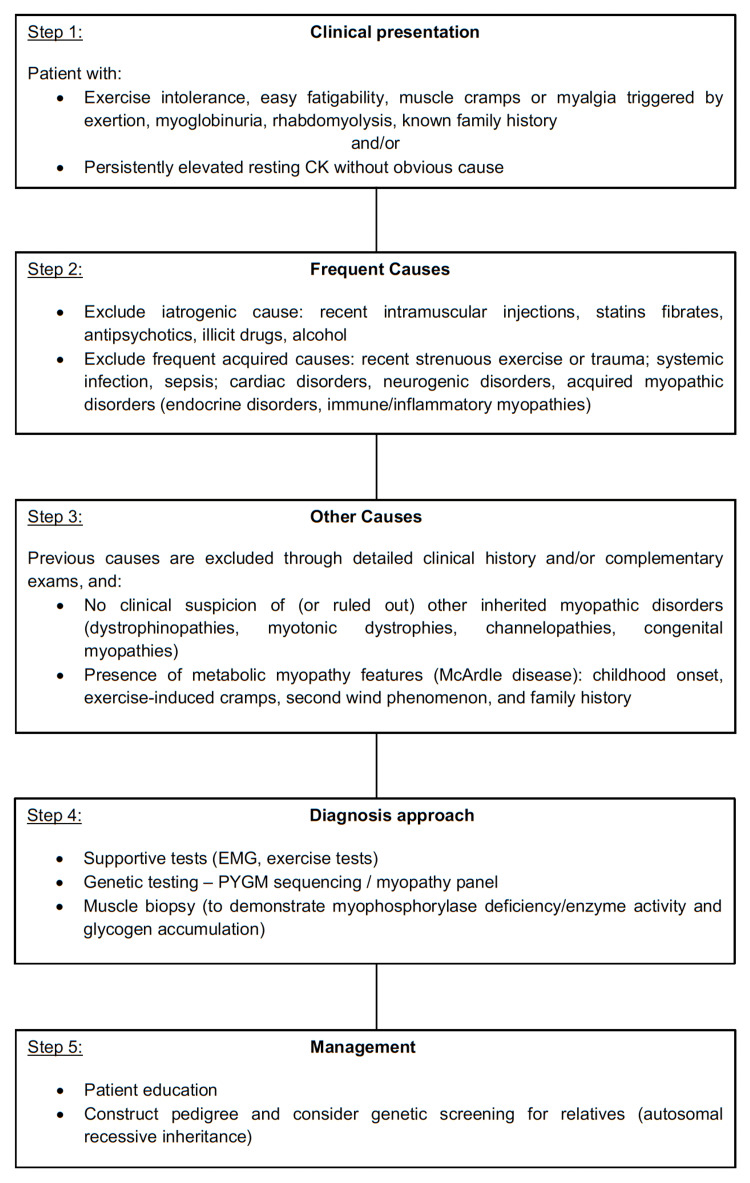
Proposed diagnostic flow chart for McArdle disease, illustrating the clinical pathway followed in this case. This flow chart illustrates the clinical pathway from initial presentation (persistent hyperCKemia and exercise intolerance) to definitive diagnosis, highlighting the role of differential diagnosis and genetic testing.

The presence of a high resting CK level is a strong indicator, but it is not specific to McArdle disease [15]. In this case, the patient's clinical history, particularly the "second wind" phenomenon and the familial component, along with the exclusion of other common causes of CK elevation, raised a high suspicion for a metabolic myopathy. The diagnostic approach followed in this case, which aligns with current clinical guidelines, is summarized in Figure 1 [7,10]. The genetic test, which identified the homozygous c.280C>T (p.Arg94Trp) variant in the PYGM gene, provided definitive confirmation, further illustrating the current diagnostic shift toward non-invasive genetic confirmation over muscle biopsy.

Furthermore, this case emphasizes the importance of proper management. Current treatment focuses on reducing symptom burden, such as frequency of cramps and fatigue, while enhancing exercise tolerance and overall quality of life, as there is no cure for this condition [8]. In this particular case, the absence of complications, such as kidney failure, simplifies the approach, focusing mainly on preventing fatigue and cramps. Dietary adjustments and supervised exercise are the bases of therapy [14]. A key strategy involves consuming carbohydrates, such as glucose or fructose, before physical activity to provide an alternative energy source, which can reduce the occurrence of cramps and fatigue [16]. Individualized low- to moderate-intensity aerobic exercise programs are also essential for improving functional capacity and preventing muscle deconditioning, further contributing to better long-term outcomes [15]. Patient education helps them recognize their limits and avoid strenuous activities that could lead to rhabdomyolysis. These non-pharmacological interventions are proven to be effective in managing the condition, although ongoing research explores gene and enzyme therapies.

## Conclusions

Prolonged fatigue is a common and non-specific symptom in everyday clinical practice and a frequent reason for referral to internal medicine. McArdle disease should be considered in the differential diagnosis specifically for patients presenting with highly suggestive features, such as lifelong exercise intolerance, exertional cramps, or persistent unexplained hyperCKemia, once more common secondary causes have been ruled out. This clinical case underscores the importance of considering rare diseases in the differential diagnosis of patients presenting with these specific chronic symptoms, particularly when other common diagnoses have been excluded. It also illustrates the significant diagnostic delay that can occur in McArdle disease, as symptoms are often normalized by the patient as part of their daily life.

Ultimately, this underscores that a detailed and meticulous clinical history remains the most fundamental tool in the diagnostic workup. Recognizing subtle patterns, such as the "second wind" phenomenon or disproportionate fatigue during anaerobic activities, is essential to identify metabolic myopathies that might otherwise be overlooked during routine assessments.
